# NGS-Based Analysis of Atypical Deep Penetrating Nevi

**DOI:** 10.3390/cancers13123066

**Published:** 2021-06-19

**Authors:** Antonella Manca, Maria Cristina Sini, Anna Maria Cesinaro, Francesca Portelli, Carmelo Urso, Maria Lentini, Roberta Cardia, Llucia Alos, Martin Cook, Sara Simi, Panagiotis Paliogiannis, Vincenzo De Giorgi, Antonio Cossu, Giuseppe Palmieri, Daniela Massi

**Affiliations:** 1 Institute of Genetic & Biomedical Research, National Research Council, 07100 Sassari, Italy; antonella.manca@cnr.it; 2 Istituto di Chimica Biomolecolare, Consiglio Nazionale delle Ricerche (CNR), 07100 Sassari, Italy; mariacristina.sini@cnr.it; 3 Azienda Ospedaliero Universitaria Policlinico Modena, 41125 Modena, Italy; cesinaro.annamaria@policlinico.mo.it; 4 Section of Pathological Anatomy, Department of Health Sciences, University of Florence, 50121 Firenze, Italy; fraportelli@gmail.com (F.P.); sara.simi@unifi.it (S.S.); 5 Dermatopathology Study Center of Florence, 50129 Florence, Italy; cylaur@tin.it; 6 Department of Human Pathology, University of Messina, 98122 Messina, Italy; lentini@unime.it (M.L.); robertacardia87@gmail.com (R.C.); 7 Hospital Clínic de Barcelona, 08036 Barcelona, Spain; lalos@clinic.cat; 8 Division of Pathology, University of Surrey, Guildford GU2 7XH, UK; m.cook@nhs.net; 9 Laboratory Quality Control Unit, University Hospital (AOU) of Sassari, 07100 Sassari, Italy; panospaliogiannis@gmail.com; 10 Dermatology Unit, Azienda USL Toscana Centro, 50122 Florence, Italy; vincenzo.degiorgi@unifi.it; 11 Department of Medical Surgical and Experimental Sciences, University of Sassari, 07100 Sassari, Italy; cossu@uniss.it; 12 Department of Biomedical Sciences, University of Sassari, 07100 Sassari, Italy; gpalmieri@yahoo.com

**Keywords:** atypical deep penetrating nevus, borderline/atypical deep penetrating nevus, deep penetrating nevus (DPN), β-catenin, next-generation sequencing (NGS)

## Abstract

**Simple Summary:**

The recent WHO classification of melanocytic tumors requires the implementation of combined phenotypic–genotypic diagnostics. For rare tumors, such as atypical deep penetrating nevi, there is insufficient information regarding genetic status, and it is not yet clear whether the observed unusual morphological cyto-architectures reflect a distinct genomic profile or are associated with an increased metastatic potential and aggressive clinical behavior. We report herein a comprehensive next-generation sequencing (NGS) analysis of a series of atypical DPNs, showing their mutational profile with some specific signatures for these rare and diagnostically challenging tumors.

**Abstract:**

Deep penetrating nevi (DPNs) are rare melanocytic neoplasms consisting of pigmented spindled or epithelioid melanocytes with a distinctive wedge-shaped configuration showing activation of the WNT pathway, with unusual cyto-architectural features. It is unclear whether they show a distinct genomic profile associated with a diverse metastatic potential. We describe herein a cohort of 21 atypical DPNs analyzed by next-generation sequencing using the Ion AmpliSeq™ Comprehensive Cancer Panel. We found that β-catenin exon 3 was mutated in 95% and MAP kinase pathway genes in 71% of the cases. Less frequent mutations were observed in HRAS (19%) and MAP2K1 (24%). Isocitrate dehydrogenases 1 (IDH1) mutations, including R132C, V178I, and S278L, were identified in 38% of cases and co-existed with BRAF/HRAS mutations. The only case with progressive nodal disease carried alterations in the β-catenin pathway and mutations in IDH1 and NRAS (codon 61). By a comprehensive mutation analysis, we found low genetic heterogeneity and a lack of significant associations between specific gene mutations and histopathological features, despite atypical features. Whether the acquisition of an NRAS or IDH1 mutation in an atypical DPN may represent a molecular evolution implying a pathway to melanoma progression should be confirmed in a larger series.

## 1. Introduction

Deep penetrating nevus (DPN) was first recognized by Seab and colleagues in 1989 [1] as an acquired dark pigmented melanocytic lesion most commonly located on the face, upper trunk, or proximal extremities of young female patients [2,3]. Histologically, DPN is characterized by spindle and epithelioid pigmented melanocytes arranged in vertically oriented loose fascicles and nests in a wedge-shaped configuration [1,4]. Due to sudden clinical onset combined with low-grade random cytologic atypia or nuclear pleomorphism, DPN may raise diagnostic doubts and be misdiagnosed as melanoma. Nuclear β-catenin immunohistochemical expression has recently been proposed as a supportive diagnostic tool [5]. Activating mutations of the β-catenin pathway, affecting CTNNB1 with rare APC mutations, and mutations in the MAPK pathway have been recognized as the major genetic abnormalities [6]. Since its initial description, DPN has been considered unique but also possibly as being a variant of, or having some relationship to, both blue nevi and Spitz tumors [1,7,8]. The plexiform spindle cell nevus, initially described as a variant of pigmented spindle cell nevus [8,9,10], has suggested a link between Spitz tumors and DPN. However, DPN has recently been confirmed as genetically distinct by the presence of activating mutations in the β-catenin and or MAP kinase pathways [6]. Furthermore, DPN is generally distinguished from Spitz tumors by the absence of kinase fusions, from blue nevi by the absence of the G-protein-activating mutations GNAQ and GNA11 [11], and from epithelioid pigmented melanocytomas by the absence of alterations in PRKAR1A and fusions of PRKCA [12].

Histopathological distinction from benign blue nevi is nosologically relevant as DPNs have been reclassified in the recent WHO Classification of Skin Tumors [7,13] in the category of “partly transformed” intermediate melanocytic tumors. This term denotes the presence of additional genetic alterations towards malignancy, but without the typical clinical, pathological, or molecular phenotype of melanoma [7,13]. Most DPNs show stable clinical behavior, although cases with locoregional metastatic potential have been reported [14,15,16,17] and morpho-molecular transformation from DPN to plexiform melanoma has occasionally been described [18].

According to the new WHO Classification [7,13], the term atypical DPN (melanocytoma) should be applied to DPNs with atypical features, including large size, asymmetry, increased cellularity with sheet-like arrangements of melanocytes, increased mitotic activity, and non-random cytological atypia. In addition, atypical DPNs with uncertain malignant potential are rare neoplasms that lack a definitive diagnosis as benign or malignant [7]. However, it remains unclear whether atypical DPN cyto-architectures reflect a distinct genomic profile or are associated with an increased metastatic potential and aggressive clinical behavior [7,8,18]. Clinical management of these lesions should entail consultation at a referral center to exclude the diagnosis of plexiform melanoma and their removal with 5–10-millimeter margins [19]. However, the utility of staging with sentinel lymph node biopsy and length of follow-up are yet to be determined [3].

The uncertainty related to atypical DPNs highlights the need to identify additional diagnostic tools. The rapid advance in next-generation sequencing (NGS) techniques offers the possibility to investigate the DPN genetic profile in depth. Here, we report an NGS analysis of a series of atypical DPNs, showing their mutational profile with some specific signatures for these rare tumors.

## 2. Materials and Methods

Electronic records of routine and consultation dermatopathology practice of the authors at the Dermatopathology Units at Florence University Hospital (Italy), Modena University Hospital (Italy), Messina University Hospital (Italy), Barcelona Hospital Clinic (Spain), and Histopathology Service, Guilford (UK), were reviewed for cases that included the terms “deep penetrating nevus” and “atypical” in the final diagnosis field. Clinical information regarding gender, age at diagnosis, and the topography of each lesion and follow-up data were obtained through the electronic medical records.

All of the diagnoses were reviewed by three expert dermatopathologists (D.M., A.M.C., and C.U.). Samples that did not meet stringent criteria, where there was a disagreement as to the diagnosis, or for which there was insufficient tissue material were excluded. The criteria for the diagnosis of typical DPN (either pure or combined forms) included primarily intradermal location, plexiform growth pattern, wedge-shaped silhouette; epithelioid melanocytes with vesicular chromatin and clear or finely pigmented cytoplasm; extension along neurovascular and/or adnexal structures; admixed melanophages, as previously described [11]. The criteria for atypical DPN were those previously set forth by WHO classification [7] and included large size (>5 mm), asymmetry, aberrant architecture (e.g., sheet-like arrangements of melanocytes or expansile nests), increased mitotic rates, and moderate to severe cytological atypia.

### 2.1. Immunohistochemistry

Immunohistochemical staining was performed on archived FFPE blocks to assess for β-catenin expression. Sections were deparaffinized in EZ prep (950–102; Ventana), and antigen retrieval was achieved by incubation with cell-conditioning solution 1 (950–124; Ventana), a Tris ethylenediaminetetraacetic acid-based buffer (pH 8.2), for 32 minutes at 100 °C. Sections were incubated with the following primary antibodies: anti β-catenin (#760–4242, mouse monoclonal, clone 14 ready to use, Ventana Medical System, Tucson, AZ, USA). The signal was developed with the UltraMap Red anti-Mouse Detection Kit (Ventana Medical Systems, Tucson, AZ, USA) in an automated Immunostainer (Ventana Discovery Ultra, Ventana Medical Systems, Tucson, AZ, USA). Sections were counterstained with hematoxylin.

β-catenin immunohistochemical expression was scored in dermal melanocytes according to the percentage of stained nuclei, regardless of any membranous or cytoplasmic staining, as previously described [5].

### 2.2. NGS Analysis

The number of samples (21 atypical DPNs) in our cohort for sequencing was limited by the availability of cases with enough residual tissue material in our archives. One dermatopathologist (D.M.) reviewed an H&E slide of each tumor sample and determined the percentage of tumor nuclei within the area to be macrodissected, with a minimum of 10% tumor nuclei present. Each specimen was macrodissected and DNA extraction was performed using a GeneRead DNA FFPE Kit, which uses special lysis conditions to overcome the inhibitory effects caused by formalin crosslinking of nucleic acids. NGS was performed in 21 FFPE samples using Ion GeneStudio S5 systems and carried out by the Ion AmpliSeq™ Comprehensive Cancer Panel, which provides a highly multiplexed target selection of 409 genes implicated in cancer research and provides a comprehensive genomic profiling solution appropriate for FFPE tissues. CCP consists of 16,000 primer pairs divided in 4 pools requiring 10 ng DNA/pool. Starting DNA and libraries were accurately quantified using a fluorescence-based quantification method, such as Qubit dsDNA HS.

The data analysis workflow was performed by automated data transfer from the Ion Torrent™ Server to the Ion Reporter Server for variant analysis; it includes result filtering, annotation, and data analysis results. Data analysis consisted of two steps: the first, performed by the Torrent Suite hosted on the Torrent Server, provides, besides base calling and sequence alignment, a first analysis of coverage metrics and basic annotation; in the second step, performed by Ion Reporter Software located in the cloud, data undergo a deeper analysis in terms of variant calling, quality evaluation, filtering, and extensive annotation. To obtain a total amount of at least 10 mutated alleles for each candidate amplicon, the following mutation selection criteria were adopted: coverage of >200 reads and frequency of mutated alleles >5% for gene amplicon.

Tumor mutation burden (TMB) values were directly calculated by the Ion Reporter™ Software using the specific panel analysis workflow.

### 2.3. Statistical Analysis

A descriptive analysis using means and standard deviations, or medians and interquartile range (IQR), was performed using MedCalc for Windows, version 15.4 64 bit (MedCalc Software, Ostend, Belgium).

## 3. Results

The clinical characteristics and outcome of the atypical DPN cases are summarized in Table 1.

Among the 21 cases included in the study, 11 (52%) were males, and the median age (IQR) was 27 (15.5–45) years. All lesions were more than 5 mm in size, with the median (IQR) diameter of the lesions being 6 (range 5.5–7.5) mm.

The most common anatomical sites were trunk and upper extremity; one case developed in the second interdigital space of the right hand and one case on the right ear. Simple excision without further surgical procedure was the main treatment in the majority of patients. Two cases underwent re-excision after initial removal; one case experienced a positive sentinel lymph node followed by axillary lymphadenectomy showing 1/20 positive right axillary lymph node. No other patients experienced local recurrence or distant metastasis, and all were alive after a median follow-up of 41 months (mean 38.1 months, range 5–226 months).

**Figure 1:**
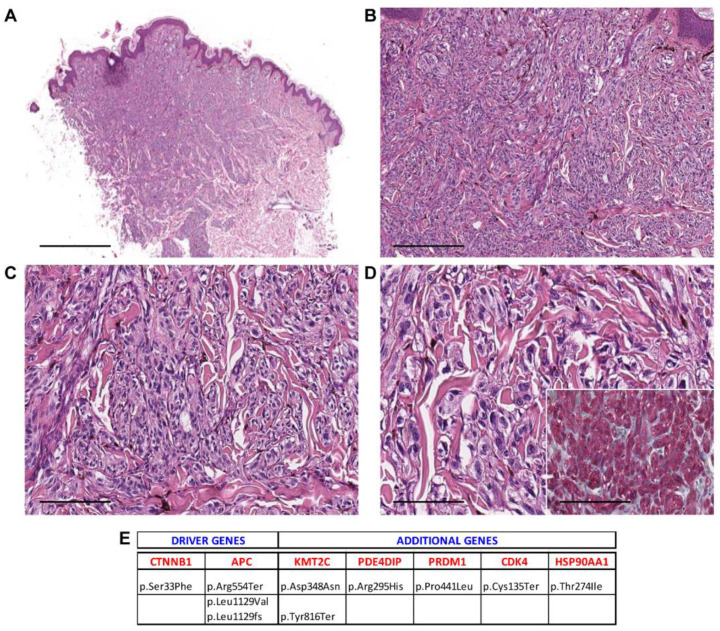
Case 2—male, 12 years old, shoulder. (**A**) Melanocytic tumor with an inverted triangle-like downward architecture in the dermis and subcutis. (40×, scale bar 500 µm); (**B**) short fascicles of spindled, ovoid, and occasionally epithelioid melanocytes (100×, scale bar 100 µm); (**C**) melanocytes intermingled with scattered melanophages (200×, scale bar 50 µm); (**D**) some melanocytes showing enlarged and hyperchromatic nuclei (400×, scale bar 25 µm). Inset: β-catenin staining shows nuclear positivity (400×, scale bar 25 µm). (**E**) Mutational profile: driver gene mutations and additional pathogenic alterations.

**Figure 2:**
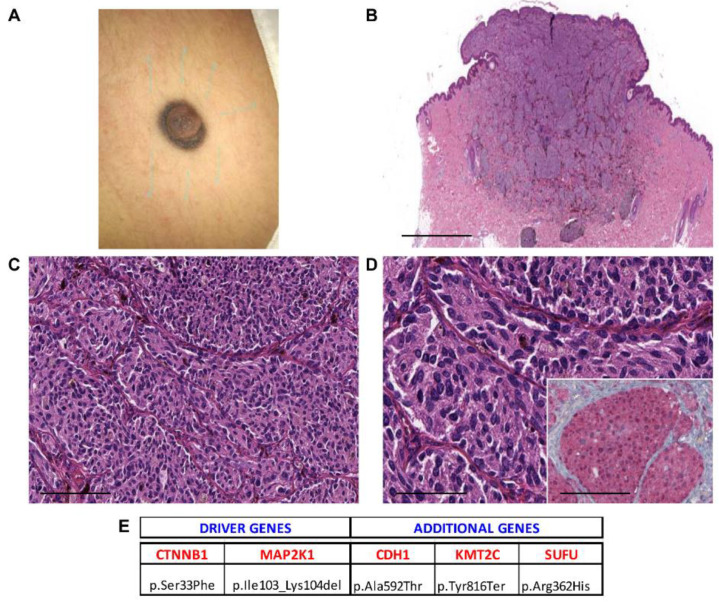
Case 3—female, 19 years old, back. (**A**) Clinical image showing a dome-shaped pigmented nodule on the back; (**B**) highly cellular dermal-based proliferation with a deep wedge-shaped configuration (40×, scale bar 500 µm); (**C**) scattered melanophages admixed with melanocytes (200×, scale bar 50 µm); (**D**) enlarged, hyperchromatic nuclei (400×, scale bar 25 µm). Inset: β-catenin immunohistochemical expression at nuclear level (400×, scale bar 25 µm). (**E**) Mutational profile: driver gene mutations and additional pathogenic alterations.

**Figure 3:**
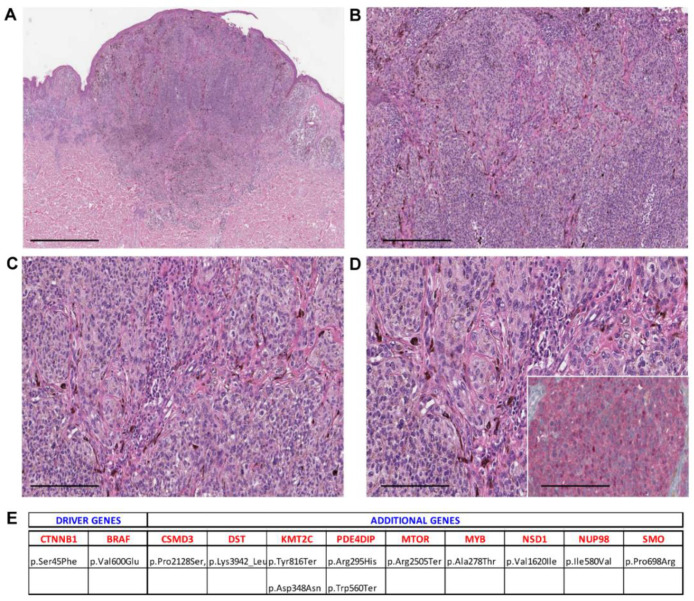
Case 9—male, 16 years old, back. (**A**) Wedge-shaped silhouette of a pigmented combined melanocytic tumor involving the dermis (40×, scale bar 500 µm); (**B**) fascicles and large aggregates of plump oval and epithelioid melanocytes combined with ovoid conventional melanocytes (100×, scale bar 100 µm); (**C**) oval melanocytes show a pale or dusty cytoplasm and contain fine melanin granules (200×, scale bar 50 µm); (**D**) oval melanocytes with hyperchromatic nuclei and pseudo-nuclear inclusions are seen intermingled with cytologically bland conventional melanocytes (400×, scale bar 25 µm). Inset: β-catenin staining shows nuclear positivity (400×, scale bar 25 µm). (**E**) Mutational profile: driver gene mutations and additional pathogenic alterations.

**Figure 4:**
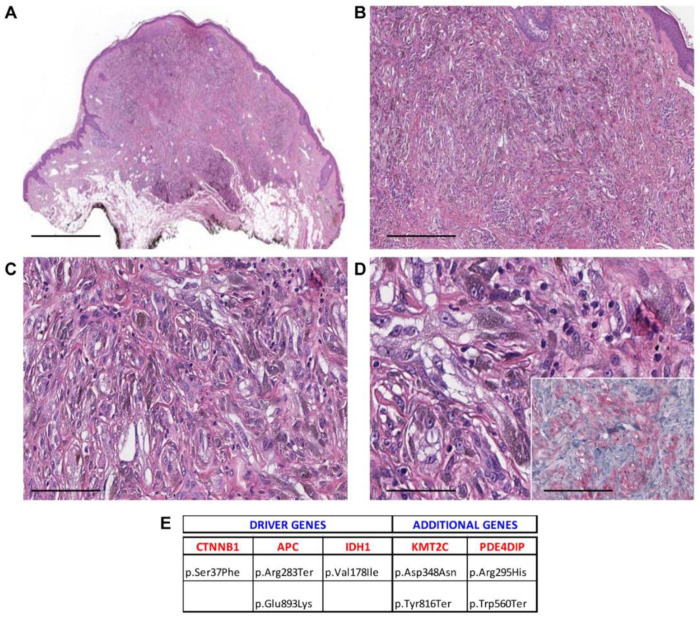
Case 16—male, 46 years old, right ear. (**A**) Asymmetric pigmented melanocytic tumor involving the dermis and subcutis and showing an expansile bulbous pattern at the base (40×, scale bar 500 µm); (**B**) the tumor is highly cellular and is composed of pigmented cells in plexiform pattern (100×, scale bar 100 µm); (**C**) several melanophages are seen within the melanocytic proliferation (200×, scale bar 50 µm); (**D**) melanocytes show a dusty-appearing pigmented cytoplasm and show enlarged nuclei (400×, scale bar 25 µm). Inset: Tumor cells show β-catenin expression at nuclear level (400×, scale bar 25 µm). (**E**) Mutational profile: driver gene mutations and additional pathogenic alterations.

**Figure 5:**
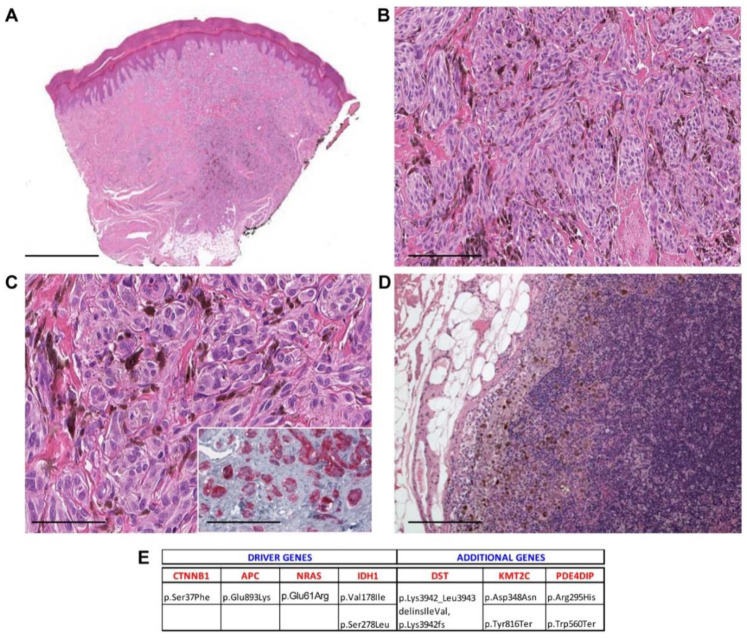
Case 17—female, 43 years old, second interdigital space, right hand. (**A**) Asymmetric melanocytic tumor involving the dermis and subcutis (40×, scale bar 500 µm); (**B**) the tumor is composed of pigmented spindle and ovoid cells in a plexiform growth pattern (200×, scale bar 100 µm); (**C**) melanocytes contain fine melanin granules, and melanophages are admixed (400×, scale bar 50 µm). Inset: Immunostaining for β-catenin shows that melanocytes retain nuclear expression for this marker (400×, scale bar 25 µm). (**D**) Representative image of hematoxylin and eosin (H&E) staining of the positive right axillary sentinel lymph node; subsequent lymphadenectomy showed an additional positive axillary lymph node (200×, scale bar 50 µm). (**E**) Mutational profile: driver gene mutations and additional pathogenic alterations.

Histopathological examination showed melanocytic lesions with features of DPN, but with enhanced cytological or architectural atypia, which exceeded the conventional DPN diagnosis. Three cases were combined (DPN1, DPN9, and DPN10) and 18/21 were pure forms. Atypical DPNs were characterized by dermal-based proliferation of enlarged pigmented spindle and epithelioid cells with scattered melanophages in a deep wedge-shaped configuration into the deep dermis (*n* = 17) or deep dermis and subcutis (*n* = 4) (Figure 1, Figure 2, Figure 3, Figure 4 and Figure 5). A cohesive pattern (sheet-like arrangement of melanocytes or expansile growth) was observed in 15/21 (75%) cases. Cytologically, the melanocytes showed moderate to marked pleomorphism in 19 cases (90%). Mitotic rates per mm^2^ were as follows: 1 mitosis/mm^2^ (*n* = 5, 24%), 2 mitoses/mm^2^ (*n* = 2, 9%), 3 mitoses/mm2 (*n* = 2, 9.5%), 4 mitoses/mm^2^ (*n* = 1, 5%), and 5/mm^2^ (*n* = 1, 5%). Deep or marginal mitoses were detected in three cases. No atypical mitoses were identified in any tumor. Half of the cases were associated with a mild to moderate lymphocytic infiltrate.

Immunohistochemical expression showed nuclear β-catenin expression in all cases (<10% stained nuclei in 14 cases, 10–30% stained nuclei in 5 cases, >30% stained nuclei in 2 cases). Cytoplasmic and membranous expression of β-catenin was also found in melanocytes, regardless of nuclear staining.

Table 2 summarizes the mutated driver genes in the cases under investigation: 1 mutated gene (*n* = 3, 14%), 2 mutated genes (*n* = 8, 38%), 3 mutated genes (*n* = 8, 38%), and 4 mutated genes (*n* = 2, 9%). The CTNNB1 gene was the most frequently mutated gene (95%), as it was found as a wild type in only one case. The APC gene was found mutated in 7/21 (33%), BRAF in 7/21 (33%), HRAS in 3/21 (14%), NRAS in 1/21 (5%), MAP2K1 in 5/21 (24%), and MAP2K2 in 1/21 (5%) cases (Table 2). Only in one case (5%) the GNAQ mutation was detected, while seven cases (33%) exhibited IDH1 mutations (Table 2, Tables S1 and S2).

Additional pathogenic mutations occurring in other genes were detected and are reported in Table 3 and Tables S1 and S2. KMT2C and PDE4DIP were the most commonly mutated genes, with 100% of samples showing mutated KMT2C and alterations in PDE4DIP being found in 67% of cases.

Atypical DPNs showed very low genetic heterogeneity for the main gene pathways, as nearly all lesions showed mutations in CTNNB1 within the β-catenin pathway and in genes belonging to the MAPK cascade (Table 4). In atypical DPNs, no statistically significant correlation between the specific gene mutation in driver genes and relevant histopathological features was demonstrated (Table 4).

## 4. Discussion

Our study provides a comprehensive mutational analysis of atypical DPNs by an extensive 409-gene tumor-specific panel, which allows highly multiplexed target selection. We identified alterations in the β-catenin gene associated with mutations in MAP kinase genes and observed frequencies in line with previous findings in typical DPN [6]. Notably, β-catenin exon 3 was mutated in 20/21 (95%) cases, while MAP kinase pathway genes exhibited mutations in 16/21 (76%). Our results support the hypothesis that activating mutations in the β-catenin gene are major driver events causing the acquisition of the DPN phenotype. Furthermore, unraveling the molecular background of the exceedingly rare DPN with atypical features, we confirmed that β-catenin mutation can be used as a diagnostic marker to assist with recognition of the DPN-associated evolutionary pathway [6].

β-catenin, a protein involved in signal transduction of the Wnt signaling pathway, encoded by the *CTNNB1* gene, is frequently altered in cancer. The main consequence of the alteration is the constitutive activation of β-catenin, which promotes cell proliferation and immunosuppression [20,21,22,23]. However, DPNs do not seem to have malignant potential, as in these lesions, the protein alteration has been associated with lack of maturation and in-depth penetration of melanocytes [6]. In addition, *CTNNB1* is not mutated in other melanocytic tumors, such as Spitz nevi, blue nevi, or melanoma.

In our series, only one case (DPN 10) resulted as wild type for β-catenin. This case harbored a high rate of copy number variations (*n* = 7.2) and presented low cellularity (15%), a condition also characterized by low frequency of the BRAF V600E allele. DPN 10 was a β-catenin-positive tumor as assessed by immunohistochemistry and was a challenging case for histopathological examination as it showed atypical features combined with a minor component of fascicles of oval- and spindle-shaped melanocytes associated with melanophages, reminiscent of epithelioid blue nevus. At the molecular level, the case also revealed a *GNAQ* mutation, a specific variant located outside the hotspot Q209 position, which has never been reported before. *GNAQ* alterations are frequently found in blue nevi, while previous studies reported that typical DPNs do not carry *GNAQ* or *GNA11* mutations and, therefore, do not belong to the family of blue nevi [11].

Previous data have shown that in DPN mutations of the MAP kinases pathway, genes arise before *CTNNB1* mutations [6]. *BRAF* V600E and *NRAS* hotspot mutations (in congenital nevi and in some acquired nevi) are frequently found in common nevi. While *HRAS* appears to be rarely mutated in common acquired or congenital nevi, mutations in this gene occur in about 10–15% of Spitz nevi, particularly in desmoplastic variants. In our cohort of atypical DPNs, *HRAS* was found to be mutated in 3/21 cases (14%), while a previous study reported *HRAS* mutations in only 6% of DPNs [11]. Specifically, 2/32 DPNs were associated with HRAS mutations, i.e., one in exon 2 (p.G13R, c.37G > C point mutation) and one in exon 3 (p.Q61L, c.182A > T point mutation) [11]. Interestingly, one of the two *HRAS*-mutated DPNs had atypical features, namely the presence of an atypical deep mitosis [11]. Thus, our results support the hypothesis that atypical DPNs may be pathogenetically related to a fraction of Spitz nevi [11].

In our samples, activating mutations in *MAP2K1* were found in 5/21 cases (24%), which is a slightly lower frequency in comparison with previous estimates (33%) [6]. We found four in-frame deletions and one single-nucleotide variation commonly reported in melanoma (COSMIC: Catalogue of Somatic Mutations in Cancer) [24]. Only one case (DPN 21) showed alterations in two contiguous codons (C125 and N126) of the *MAP2K2 (MEK2)* gene. One of these, the C125S variant, was previously identified in melanoma patients who developed resistance to *BRAF* inhibitors [25,26]. In a recent study, MAP2K1 alterations were reported in neoplasms with heterogeneous cell morphology, including melanocytic tumors with Spitz cytomorphology, DPN-like architecture with a plexiform pattern, and heavy melanization [27].

Isocitrate dehydrogenases 1 (*IDH1*) mutations—including R132C, V178I, and S278L—were identified with a relatively high frequency in our series (8/21 cases; 38%), co-existing with *BRAF/HRAS* mutations. Among these variants, only R132C has been recognized to have a pathogenic effect [28]. IDH mutation-induced hypermethylation may lead to suppression of cellular differentiation. Such mutations have been reported in a variety of cancers, including glioma, melanoma, and cholangiocarcinoma [28,29]. Hypermethylation promoted by these mutations seems to act as a favorable prognostic marker in glioma [28]. These metabolic enzymes normally convert isocitrate into α-ketoglutarate; when modified in key arginine residues that normally bind isocitrate substrate, the mutant enzyme causes a radical change in enzymatic activity by converting α-ketoglutarate into D-2-hydroxyglutarate (D2HG). D2HG acts as a competitive inhibitor of other enzymes that require α-ketoglutarate as a cofactor, including certain DNA demethylases, leading to genomic CpG hypermethylation and globally altered transcription.

In addition to CTNNB1 and MAP kinase components, sequence variations were found in additional genes, with *KMT2C* and *PDE4DIP* being the most frequently altered. However, all of these additional variants are classified as “likely benign” in the VARSOME database [30]. The large size of these two genes may account for the high frequency of alterations detected. The low-density lipoprotein receptor-related protein 1b (*LRP1B*) gene was found mutated in about one-fifth (4/21; 19%) of cases in our series. *LRP1B* has been reported to exert tumor suppressor activity, negatively regulating β-catenin/TCF signaling [31]. Interestingly, human cancers carrying *LRP1B* mutations seem to increase their responsiveness to the immune checkpoint inhibitors [32].

Our study demonstrated that atypical DPNs occur in young age, with no sex predilection, and any anatomical site can be affected. Despite the worrying features at histopathological examination, none of these cases were associated with fatal outcome. Only 1/21 cases experienced regional (lymphnodal) disease progression (DPN 17) from a tumor that developed in the second interdigital space of the right hand. At the genetic level, besides alterations in the β-catenin pathway, the tumor carried mutations in *IDH1* and *NRAS* (codon 61) genes. In the context of DPN, *NRAS* mutations have been reported thus far in sporadic observations, including a combined nevus with a DPN component and a case of atypical DPN progressing to melanoma [33,34]. In addition, a subset of melanomas with *IDH1* mutations that are significantly associated with co-existing *NRAS* mutations has been described [29].

A recent literature review summarizing results on 355 DPN patients and 48 atypical/borderline DPN patients reported a total of five recurrences (2 in DPN and 3 in borderline DPN group) and three metastases in the borderline DPN group [35]. Due to their rarity, the biological significance of lymphnodal deposits is still unclear, although it has been suggested that for those cases classified as borderline DPN, there is a significant risk for regional lymphnode disease and progression to overt melanoma [18].

## 5. Conclusions

In conclusion, the mutational landscape of 21 DPN cases with atypical features, obtained by an NGS-based comprehensive mutation analysis, indicated a low genetic heterogeneity in these lesions and a lack of substantial associations between specific gene mutations and particular histopathological features. Whether the acquisition of an *NRAS* or *IDH1* mutation in an atypical DPN may represent a molecular evolution, implying a road to progression toward a different biological behavior, should be confirmed in a larger series.

## Figures and Tables

**Table 1:** Clinical features and outcome of 21 atypical DPN cases.

Case	Sex/Age (Years)	Location	Size (mm)	Treatment	Progression	Status (Follow-Up, Months)
1	F/19	Back	15	Excision	No	Alive, NED, 72 months
2	M/12	Right shoulder	6	Excision	No	Alive, NED, 226 months
3	F/19	Back	7	Excision	No	Alive, NED, 9 months
4	M/25	Right forearm	5	Excision	No	Alive, NED, 120 months
5	M/3	Right thigh	6	Excision	No	Alive, NED, 43 months
6	F/54	Left arm	5	Excision	No	Alive, NED, 31 months
7	M/43	Neck	7	Excision	No	Alive, NED, 28 months
8	F/56	Back	8	Excision	No	Alive, NED, 58 months
9	M/16	Back	15	Excision	No	Alive, NED, 41 months
10	M/29	Back	5	Excision	No	Alive, NED, 78 months
11	F/44	Abdomen	7	Excision	No	Alive, NED, 44 months
12	F/13	Back	6	Excision	No	Alive, NED, 28 months
13	F/14	Abdomen	18	Excision	No	Alive, NED, 69 months
14	F/29	Right shoulder	7	Excision followed by re-excision	No	Alive NED, 15 months
15	M/38	Lumbar region	6	Excision	No	Alive, NED, 138 months
16	M/46	Right ear	5	Excision	No	Alive, NED, 155 months
17	F/43	Second interdigital space, right hand	7	Excision followed by re-excision + SLNB + complete right axillary lymphadenectomy	Positive SLNB and 1/20 positive right axillary lymph node	Alive, NED, 72 months
18	M/21	Left arm	10	Excision	No	Lost to follow-up
19	M/15	Left shoulder	6	Excision	No	Lost to follow-up
20	M/39	Lumbar region	5	Excision	No	Alive, NED, 14 months
21	F/30	Left arm	6	Excision	No	Alive, NED, 5 months

(F: female; M: male; SLNB: sentinel lymph node biopsy; NED: no evidence of disease).

**Table 2:** Driver gene pathogenic mutations detected in 21 atypical DPNs.

Case	TMB	Driver Genes Mutated	Pathogenic Variants	β-Catenin Pathway		MAPK Pathway			Other	
1 *	14.3	3	3	CTNNB1	APC	BRAF				
2	234.6	2	3	CTNNB1	APC					
3	13.2	2	2	CTNNB1				MAP2K1		
4	21.7	1	1	CTNNB1						
5	7.7	1	1	CTNNB1						IDH1
6	21.4	3	5	CTNNB1		BRAF				IDH1
7	16.0	3	3	CTNNB1	APC		HRAS			
8	15.9	3	3	CTNNB1				MAP2K1		IDH1
9 *	15.6	2	2	CTNNB1		BRAF				
10 *	34.6	2	2			BRAF			GNAQ	
11	11.7	1	1	CTNNB1						
12	12.5	2	2	CTNNB1			HRAS			
13	60.1	3	3	CTNNB1	APC	BRAF				
14	14.21	3	3	CTNNB1		BRAF				IDH1
15	16.08	3	3	CTNNB1	APC		HRAS			
16	12.64	3	4	CTNNB1	APC					IDH1
17	66.67	4	5	CTNNB1	APC		NRAS			IDH1
18	14.08	2	2	CTNNB1				MAP2K1		
19	15.7	2	2	CTNNB1				MAP2K1		
20	16.75	4	4	CTNNB1		BRAF		MAP2K1		IDH1
21	19.11	2	2	CTNNB1				MAP2K2		

* Combined lesions upon histopathological examination. TMB: tumor mutational burden.

**Table 3:** Further pathogenic mutations detected in 21 atypical DPNs.

Case	Other Mutated Genes	Pathogenic Variants	Mutated Genes
1*	3	5	KMT2C, PDE4DIP, MLH1
2	5	5	KMT2C, PDE4DIP, CDK4, HSP90AA1, PRDM1
3	3	3	KMT2C, CDH1, SUFU
4	3	3	KMT2C, CBL, ERBB4
5	4	6	KMT2C, PDE4DIP, ADAMTS20, IGF2
6	6	7	KMT2C, LRP1B, CDH1, ERBB2, IGF2, PAK3
7	4	5	KMT2C, PDE4DIP, KIT, IGF2
8	4	5	KMT2C, PDE4DIP, IGF2, PER1
9 *	10	11	KMT2C, PDE4DIP, CSMD3, DST, MTOR, MYB, NSD1, NUP98, PER1, SMO
10 *	1	1	KMT2C
11	2	3	KMT2C, PDE4DIP
12	1	1	KMT2C
13	3	2	KMT2C, PDE4DIP, IGF2
14	6	6	KMT2C, PDE4DIP, LRP1B, ATM, PER1, RALGDS
15	4	5	KMT2C, PDE4DIP, CSF1R, PTCH1
16	2	4	KMT2C, PDE4DIP
17	3	5	KMT2C, PDE4DIP, DST
18	3	2	KMT2C, PDE4DIP, KDR
19	6	7	KMT2C, PDE4DIP, LRP1B, KDR, AXL, PTCH1
20	3	4	KMT2C, PDE4DIP, ASXL1
21	6	20	KMT2C, PDE4DIP, GUC1A2, RNF213, SYNE1, TAF1L

* Combined lesions upon histopathological examination.

**Table 4:** Correlations between histopathological features and driver gene mutations in 21 atypical DPNs.

Histopathological Feature	Cases	CTNNB1	APC	BRAF	HRAS	NRAS	MAP2K1	MAP2K2	IDH1
**Mitotic rate/mm^2^**									
=0	10	10	3	3	2	0	3	0	4
≥1	11	10	4	4	1	1	2	1	3
**Deep/marginal mitoses**									
=0	18	17	5	7	3	0	4	1	5
≥1	3	3	2	0	0	1	1	0	2
**Size**									
<6.5 mm	11	10	3	3	2	0	2	1	4
≥6.5 mm	10	10	4	4	1	1	3	0	3
**Extension into the subcutis**									
Absent	17	16	4	7	3	0	4	1	5
Present	4	4	3	0	0	1	1	0	2
**Pleomorphism**									
Absent	2	2	2	1	0	0	0	0	0
Present	19	18	5	6	3	1	5	1	7
**Inflammatory infiltrate**									
Absent	10	9	5	3	2	1	1	0	2
Present	11	11	2	4	1	0	4	1	5

## Data Availability

Supporting data will be available upon request.

## Supplementary Materials

The following are available online at https://www.mdpi.com/article/10.3390/cancers13123066/s1, Table S1: Oncoplot of gene mutation distribution; each column represents a sample and each row a different gene, Table S2: Table representation of specific gene variants in detail; each column represents a sample and each row a different gene.
