# Supplementary material for: NGS-Based Analysis of Atypical Deep Penetrating Nevi
Source: Cancers (Basel). 2021 Jun 19;13(12):3066. doi: 10.3390/cancers13123066 (PMC8234376; doi:10.3390/cancers13123066)
Supplement: Supplementary file 1 [file cancers-13-03066-s001.zip › cancers-1204772-supplementary.pdf]

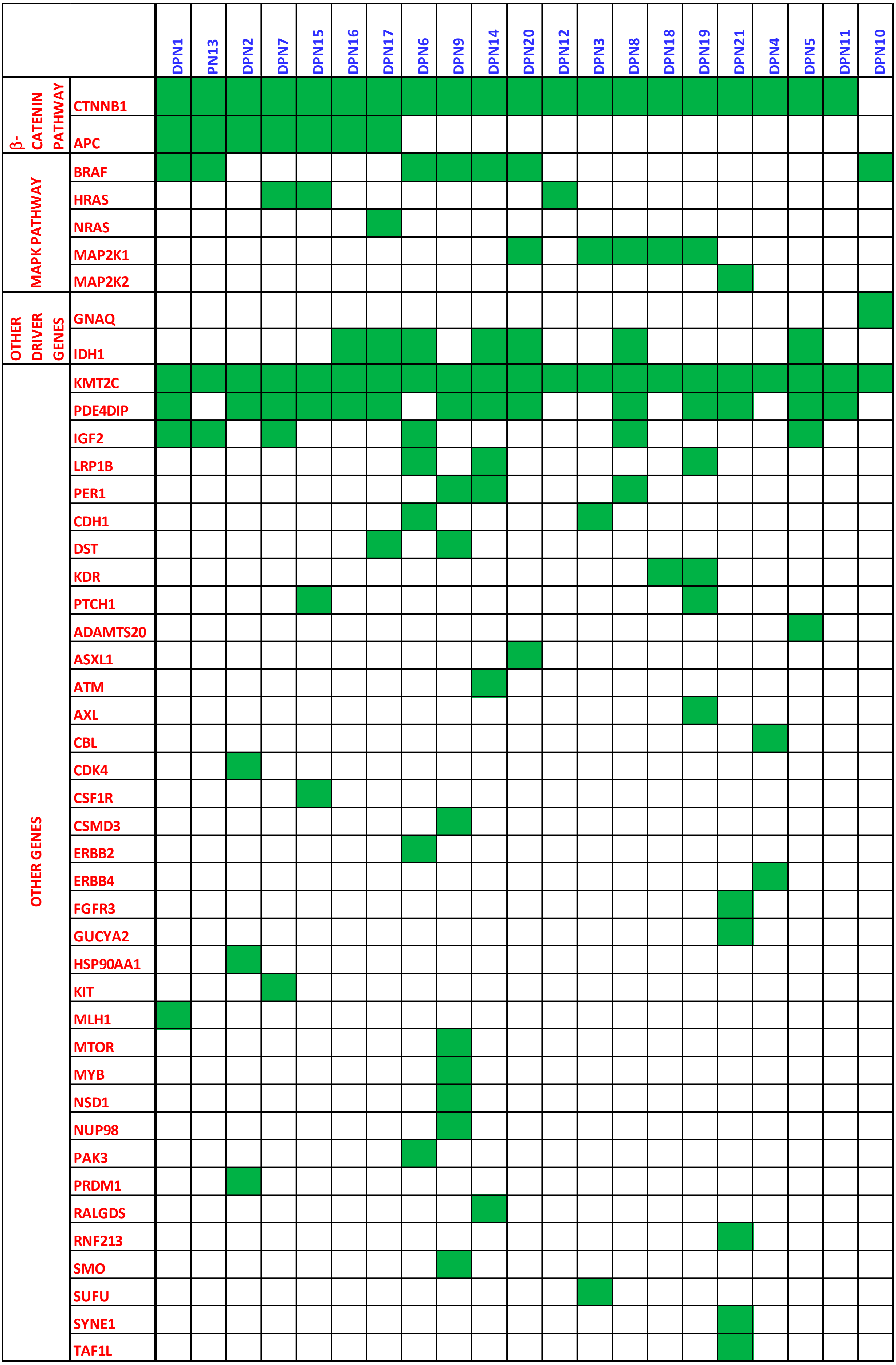

S1. Oncoplot of gene mutation distribution; each column represents a sample and each row a different gene.

		DPN1	DPN2	DPN3	DPN4	DPN5	DPN6	DPN7	DPN8	DPN9	DPN10	DPN11	DPN12	DPN13	DPN14	DPN15	DPN16	DPN17	DPN18	DPN19	DPN20	DPN21
β–CATENIN PATHWAY	CTNNB1	S45F	S33F	S33F	S37F	G34E	G34V	S37F	S37F	S45F		S33F	S37F	S37F	S37F	D32H	S37F	S37C	S37F	G34K	H36F	S33F
	APC	L1129V	L1129V<br>S331L<br>R554*<br>S2129L					L1129V						S940L<br>L1129V		R805*	R283*<br>E893K	E893K				
MAPK PATHWAY	BRAF	V600E					V600K<br>V600M			V600E	V600E			V600E	R389C						V600K	
	NRAS																	Q61R				
	HRAS							G13R					G13S			Q61K						
	MAP2K1			I103_K104<br>del					I103_K104<br>del										I103_K104<br>del	I103_K104<br>del	P124S	
	MAP2K2																					C125S<br>N126D
	GNAQ										T85M											
	IDH1					R132C	c.520+2T>C<br>R132C		V178I						V178I		V178I	S278L<br>V178I			R132C	
OTHER GENES	KMT2C	Y816*	Y816*<br>D348N	Y816*	Y816*	Y816*<br>D348N	Y816*<br>D348N	Y816*	Y816*	Y816*<br>D348N	Y816*	Y816*<br>D348N	Y816*	Y816*	Y816*<br>D348N	Y816*<br>D348N	Y816*<br>D348N	Y816*<br>D348N	Y816*	Y816*	Y816*<br>D348N	Y816*<br>D348N
	PDE4DIP	R622*<br>W560*	R295H			W560*<br>R295H		W560*<br>R295H	W560*<br>R295H	W560*<br>R295H		W560*			R295H	R295H	W560*<br>R295H	W560*<br>R295H		W560*<br>R295H	W560*	R25L<br>S275L<br>R295H<br>E410V<br>S536T<br>W560*<br>R681H<br>A1066T<br>K1266E<br>A1742S<br>R1867C
	IGF2	G226R<br>G226fs				G226R<br>G226fs	G226R<br>G226fs	G226R<br>G226fs	G226R<br>G226fs					G226R<br>G226fs								
	LRP1B						A3308V								E668K					R790Q		
	PER1								P766L<br>P766fs													
	CDH1			A592T			A592T															
	DST									K3942_L3<br>943<br>delinsIV<br>K3942fs								K3942_L3<br>943<br>delinsIV<br>K3942fs				
	KDR																		G539R	S264F		
	PTCH1															N1392K<br>R1394fs				G38E		
	ADAMTS20					V1166I																
	ASXL1																				G704R	
	ATM														S1691R							
	AXL																			G765E		
	CBL				E862K																	
	CDK4		C135*																			
	CSF1R															E705K						
	CSMD3									P2128S<br>P2128fs												
	ERBB2						G1015E															
	ERBB4				P747L																	
	FGFR3																					
	GUCYA2																					
	HSP90AA1		T274I																			
	KIT							E490V														
	MLH1	R325Q																				
	MTOR									R2505*												
	MYB									A278T												
	NSD1									V1620I												
	NUP98									I580V												
	PAK3						R455Q															
	PRDM1		P441L																			
	RALGDS														L242I							
	RNF213																					
	SMO									P698R												
	SUFU			R362H																		
	SYNE1																					
	TAF1L																					

S2. Table representation of specific gene variants in detail; each column represents a sample and each row a different gene.
